# Temporal Alignment of Wastewater Signals with Clinical Indicators of Respiratory Illness Postpandemic, Texas, USA

**DOI:** 10.3201/eid3213.260141

**Published:** 2026-08

**Authors:** Kaiming Bi, Micaela Sandoval, Thuy Nguyen, Isrrael Perez, Lopita Ghosh, Trudy Krause, Cecilia Ganduglia Cazaban, Remy Pasco, Lauren Meyers, Fuqing Wu, Janelle Rios, Michael Tisza, Justin Clark, Anthony Maresso, Eric Boerwinkle, Cici Bauer

**Affiliations:** University of Texas Health Science Center at Houston, Houston, Texas, USA (K. Bi, M. Sandoval, T. Nguyen, I. Perez, L. Ghosh, T. Krause, C. Ganduglia Cazaban, F. Wu, J. Rios, E. Boerwinkle, C. Bauer); Texas Epidemiologic Public Health Institute, Houston (K. Bi, M. Sandoval, T. Nguyen, F. Wu, J. Rios, E. Boerwinkle, C. Bauer); University of Colorado Boulder, Boulder, Colorado, USA (R. Pasco); The University of Texas at Austin, Austin (L. Meyers); Baylor College of Medicine, Houston, Texas, USA (M. Tisza, J. Clark, A. Maresso)

**Keywords:** Wastewater surveillance, influenza, respiratory syncytial virus, Covid-19, viruses, pandemic, respiratory infections, Texas, United States

## Abstract

In the COVID-19 postpandemic era, declining clinical testing for SARS-CoV-2 has led to the exploration of complementary early detection methods. We evaluated metagenomic wastewater-based epidemiology (WBE) in Texas, USA, by comparing viral signals with the National Syndromic Surveillance Program tracked emergency department visits and Texas All-Payer Claims Database insurance claims across several Texas counties during 2022–2024. By analyzing SARS-CoV-2, influenza, and respiratory syncytial virus, we found moderate-to-strong temporal correlations between wastewater and clinical indicators. Influenza showed the most stable associations (*r*<0.98), whereas SARS-CoV-2 signals generally preceded clinical metrics. Respiratory syncytial virus exhibited higher geographic and temporal heterogeneity, reflecting differences in clinical data capture. Despite sampling frequency and geographic alignment challenges, metagenomic WBE consistently tracked community trends. Our findings suggest WBE can offer valuable situational awareness and a resilient complement to clinical surveillance, supporting public health preparedness in an evolving respiratory disease landscape.

Respiratory viruses, specifically influenza, respiratory syncytial virus (RSV), and SARS-CoV-2, cause a broad spectrum of illness (*1*,*2*), from mild symptoms to severe pneumonia and acute respiratory failure. Those pathogens are primary drivers of global morbidity and mortality, placing immense strain on healthcare systems (*2*). Worldwide, influenza causes ≈400,000 deaths every year (*3*), and the COVID-19 pandemic resulted in over 7.1 million deaths by early 2025 (*4*). Historical outbreaks underscore the critical need for robust, diversified surveillance to track transmission and guide public health interventions (*5*).

Traditional public health surveillance provides the foundation for monitoring those trends. In the United States, the National Syndromic Surveillance Program (NSSP) offers near–real-time indicators of community respiratory activity by tracking emergency department (ED) visits (*6*). However, ED data primarily reflect more severe cases and specific care-seeking behaviors, potentially serving as an incomplete proxy for total community-level infection rates (*7*,*8*). Furthermore, operational differences and reporting inconsistencies can create artificial fluctuations in temporal trends (*9*). To address those gaps, alternative data streams are needed. Administrative health insurance claims data represent a promising but underutilized resource; they capture a broad range of clinical encounters beyond the ED, offering a high-resolution view of healthcare utilization and disease across diverse demographics (*10*).

Wastewater-based epidemiology (WBE) has emerged as a powerful, noninvasive complement to clinical data (*11*). Modern virome sequencing-based WBE enables the simultaneous quantification and variant characterization of thousands of viruses from a single sample (*12*,*13*). Although WBE was widely adopted during the COVID-19 pandemic, few studies have rigorously evaluated how metagenomic viral detection in wastewater sequencing data for viruses with pandemic potential, other than SARS-CoV-2, correlates with clinical metrics in the postpandemic context.

We leveraged viral metagenomic sequencing data from a statewide monitoring initiative in Texas, USA, to evaluate the utility of WBE for ongoing respiratory disease monitoring. We compared wastewater signals for SARS-CoV-2, influenza, and RSV across 10 counties to both NSSP syndromic surveillance and health insurance claims data. Our analysis focuses on the strength and consistency of those correlations and the lead and lag timing of wastewater signals relative to clinical indicators across diverse sites and seasons. By integrating insurance claims as a primary comparative metric, we evaluate the potential of this administrative data source to fill gaps in traditional surveillance, ultimately enhancing situational awareness and supporting broader public health monitoring efforts.

## Method 

### Study Region and Population

We conducted a hybrid surveillance validation study across 10 Texas counties (Cameron, Chambers, El Paso, Fort Bend, Harris, Hays, Lubbock, Travis, Wichita, and Williamson) in the Texas Wastewater Environmental Biomonitoring (TexWEB) program (*12*). During June 2022–June 2024, we collected wastewater samples near-weekly and sequenced for SARS-CoV-2, influenza (A and B), and RSV. Coverage for El Paso, Fort Bend, Harris, and Lubbock spans the full 24-month period, whereas data for the remaining counties were available for June 2023–June 2024. Those jurisdictions represent diverse demographic profiles, including major metropolitan areas like Houston (Harris County) (Table 1). 

**Table 1:** County population estimates and number of wastewater-based surveillance sites, Texas, USA, 2024

County	Estimated population	No. sampling sites
Cameron	431,874	2
Chambers	31,475	2
El Paso	875,784	4
Fort Bend	958,434	6
Harris	5,009,302	8
Hays	292,029	3
Lubbock	278,902	2
Travis	1,363,767	3
Wichita	137,363	1
Williamson	727,480	3

### Wastewater Sample Collection 

We obtained wastewater samples through the TexWEB network, which monitors 41 publicly owned treatment sites across Texas (*12*). This study included 23 sites within the TexWEB network that maintained near-weekly sampling and consistent sequencing-based reporting for SARS-CoV-2, influenza, and RSV during June 2022–June 2024 (Appendix Table 1). We report viral abundance as reads per million filtered (RPMF), a metagenomic metric that captures a genomewide, variant-inclusive signal. Unlike traditional quantitative PCR–based concentrations (e.g., gene copies per liter), this metagenomic approach enables the simultaneous detection of multiple pathogens and remains resilient to primer-mismatch errors caused by viral evolution, although it measures relative abundance rather than absolute concentration (*14*–*16*). For counties with multiple sampling sites, we calculated a county-level RPMF by using a population-weighted average to ensure a stable comparison with NSSP records and administrative insurance claims.

### Validation Clinical Outcome Datasets

We used 2 complementary clinical data sources to validate wastewater signals: the NSSP and the Texas All-Payer Claims Database (TX-APCD). The NSSP Electronic Surveillance System for the Early Notification of Community-based Epidemics provides near–real-time data from participating Texas facilities, capturing ≈90% of healthcare facilities in the Houston region (*17*), enabling the detection of rapid shifts in community respiratory activity (*6*). We derived pathogen-specific counts for COVID-19, influenza, and RSV (June 2022–June 2024) by using queries from the Electronic Surveillance System for the Early Notification of Community-based Epidemics that integrate chief complaints with discharge diagnoses, including clinical term codes from the International Classification of Diseases, 10th Revision, Clinical Modification (ICD-10-CM); International Classification of Diseases, 9th Revision, Clinical Modification; and Systematized Nomenclature of Medicine (*18*).

The TX-APCD, managed by UTHealth Houston, offers high-resolution diagnostic confirmation across commercial, Medicaid, and Medicare advantage payers, covering 74% of insured persons from Texas (*19*). We extracted ED claims through June 2024, identifying encounters through ICD-10-CM diagnosis codes supplemented by current procedural terminology and national drug codes for laboratory testing and treatment (Table 2; Appendix Tables 2, 3). Although the NSSP excels in timeliness, the TX-APCD provides superior diagnostic specificity across a broad payer mix.

**Table 2:** Diagnosis and treatment codes used to identify respiratory diseases in the Texas All-Payer Claims Database emergency department claims data, Texas, USA, 2024*

Respiratory disease	Coding system	Codes used
COVID-19	ICD-10	U07.1, U09, U09.9, U04.9, J12.82
	CPT	J0248
	NDC	11 COVID-19–related NDC codes (Appendix Table 2†)
Influenza	ICD-10	J09–J11
	NDC	65 influenza-related NDC codes (Appendix Table 3†)
Respiratory syncytial virus	ICD-10	B97.4, J12.1, J20.5, J21.0

*CPT, current procedural terminology; ICD-10, International Classification of Diseases, 10th Edition; NCD, national drug codes. †Appendix

We aggregated claims to weekly counts by residential postal (ZIP) code. To differentiate new infection episodes from follow-up care, we defined a new case by using a 30-day disease-free interval for COVID-19 and influenza and a 45-day interval for RSV. Those thresholds are consistent with established epidemiologic standards for administrative data to minimize duplicate counting of single illness episodes (*20*–*22*). That dual-source approach pairs the broad, population-level coverage of syndromic surveillance with the diagnostic specificity of administrative claims to provide a robust validation of metagenomic wastewater metrics.

### Statistical Analysis

We interpolated wastewater viral signals by RPMF to daily resolution by using natural cubic splines to enable comparison with clinical data (*23*). As a global smoothing method, this spline interpolation uses information from both past and future observations; consequently, we can interpret the resulting temporal relationships as descriptive associations rather than strictly real-time predictive leads. For clinical data, we smoothed daily ED visits by using a trailing 7-day moving average (right-aligned) to reduce day-of-week variability while ensuring only past information was used for each data point.

We conducted a time-lagged cross-correlation analysis to evaluate the temporal relationships between wastewater signals and clinical indicators. For the daily NSSP data, we assessed correlations across a +21-day window. For the TX-APCD administrative claims, which are reported at a lower temporal resolution, we compared weekly aggregated wastewater data to weekly claims data using a +3-week window. We used Pearson correlation coefficients to quantify those associations, because that metric directly captures the linear relationship and signal intensity (e.g., peak magnitude) between viral concentrations and visit rates. We adjusted CIs for autocorrelation to ensure valid inference.

To account for evolving viral dynamics and spatial heterogeneity, we performed separate analyses for each county and stratified the results by dominant variant or respiratory season. For SARS-CoV-2, we defined 2 major periods: June 2022–June 2023 (XBB variant dominance) and June 2023–June 2024 (emergence of EG.5, HV.1, and JN.1 variants). We stratified influenza and RSV analyses by the 2022–23 and 2023–24 respiratory seasons (*24*). Those stratifications enabled us to evaluate the consistency of wastewater signal associations across shifts in viral lineages and seasonal patterns.

## Results

**Figure 1:**
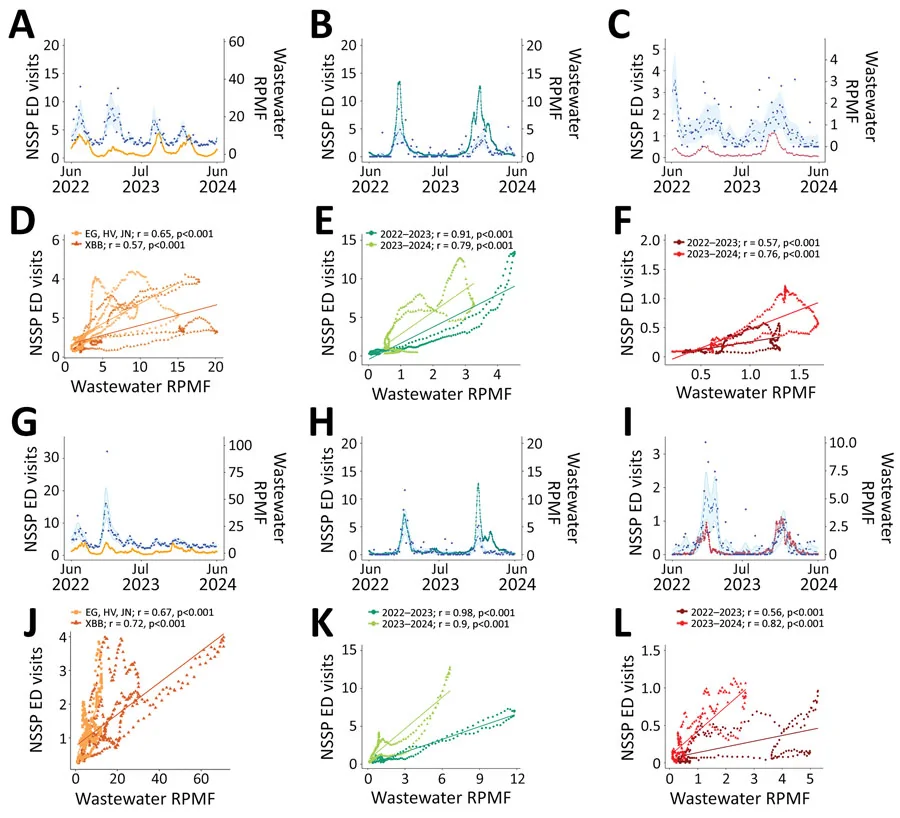
Association between daily wastewater signals (RPMF) and daily NSSP ED visits (per 100,000 population) for SARS-CoV-2, influenza, and respiratory syncytial virus (RSV) in Harris and El Paso Counties, Texas, June 2022–June 2024. A–C) Harris County interpolated daily time series of RPMF values versus NSSP ED visits for SARS-CoV-2 (A), influenza (B), and RSV (C). Solid lines represent ED visits; dashed lines represent RPMF data. D–F) Daily RPMF values versus NSSP ED visits for SARS-CoV-2 (D), influenza (E), and RSV (F). Triangles represent single-day observations. G–I) El Paso County interpolated daily time series of RPMF values versus NSSP ED visits for SARS-CoV-2 (G), influenza (H), and RSV (I). Solid lines represent ED visits; dashed lines represent RPMF data. J–L) Daily RPMF values versus NSSP ED visits for SARS-CoV-2 (J), influenza (K), and RSV (L). Triangles represent single-day observations. Corresponding Pearson correlation coefficients (*r*) and p values are shown. ED, emergency department; NSSP, National Syndromic Surveillance Program; RPMF, reads per million filtered.

**Figure 2:**
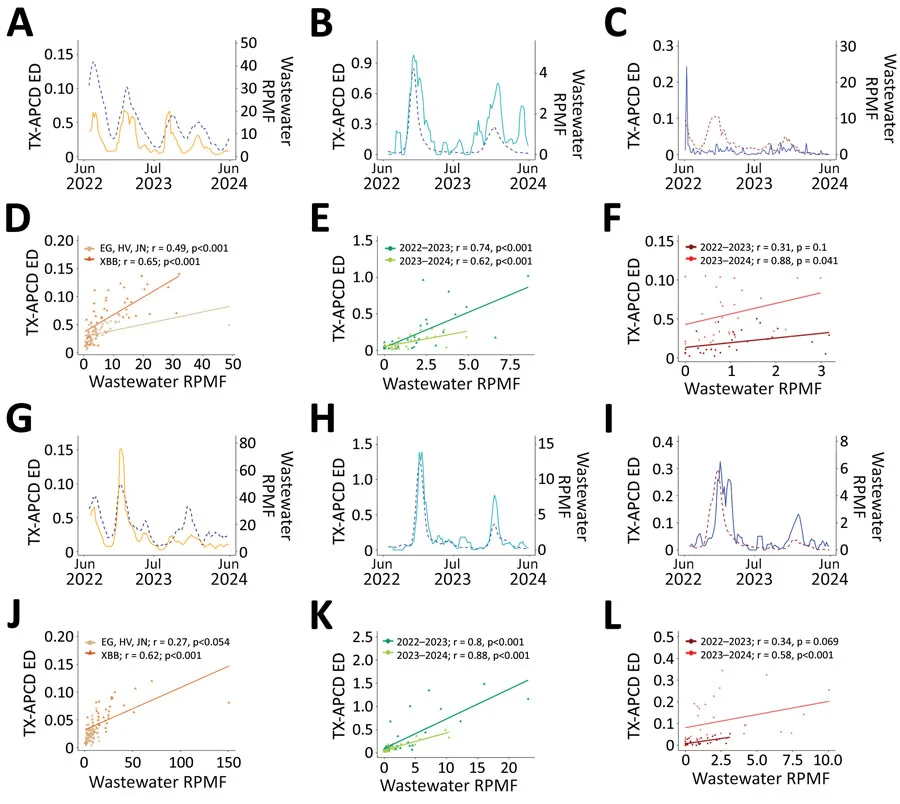
Association between weekly wastewater signals (RPMF) and weekly TX-APCD ED claims for SARS-CoV-2, influenza, and RSV in Harris and El Paso Counties, Texas, June 2022–June 2024. A–C) Harris County weekly trends in wastewater RPMF values versus TX-APCD ED visits for SARS-CoV-2 (A), influenza (B), and RSV (C). Solid lines represent TX-APCD claims; dashed lines represent RPMF data. D–F) Weekly RPMF values versus NSSP ED visits for SARS-CoV-2 (D), influenza (E), and RSV (F). Triangles and diamonds represent single-week observations. G–I) El Paso County interpolated daily time series of RPMF values versus TX-APCD ED visits for SARS-CoV-2 (G), influenza (H), and RSV (I). Solid lines represent TX-APCD claims; dashed lines represent RPMF data. J–L) Weekly RPMF values versus NSSP ED visits for (J) SARS-CoV-2, (K) influenza, and (L) RSV. Triangles and diamonds represent single-week observations. Corresponding Pearson correlation coefficients (*r*) and p values are shown. ED, emergency department; RPMF, reads per million filtered; TX-APCD, Texas All-Payer Claims Database.

During June 2022–June 2024, wastewater RPMF values demonstrated positive correlations with NSSP ED visits at 0 lag across SARS-CoV-2, influenza, and RSV (Figure 1). In Harris and El Paso counties, Pearson correlation coefficients (*r*) for influenza were strong over the full study period (*r* = 0.79–0.98). For those 2 counties, SARS-CoV-2 correlations were moderate to strong (*r* = 0.57–0.72), whereas RSV associations revealed greater seasonal variability (*r* = 0.57–0.82). Comparisons between weekly wastewater signals and TX-APCD claims revealed a wider range of associations (Figure 2). Although influenza showed strong concordance in both Harris (*r* >0.62) and El Paso (*r* >0.8) counties, RSV correlations varied widely, including weak associations in Harris County. Those discrepancies were highlighted by divergent peak timings; a June 2022 RSV surge was evident in wastewater and NSSP ED visits but absent in TX-APCD claims.

**Figure 3:**
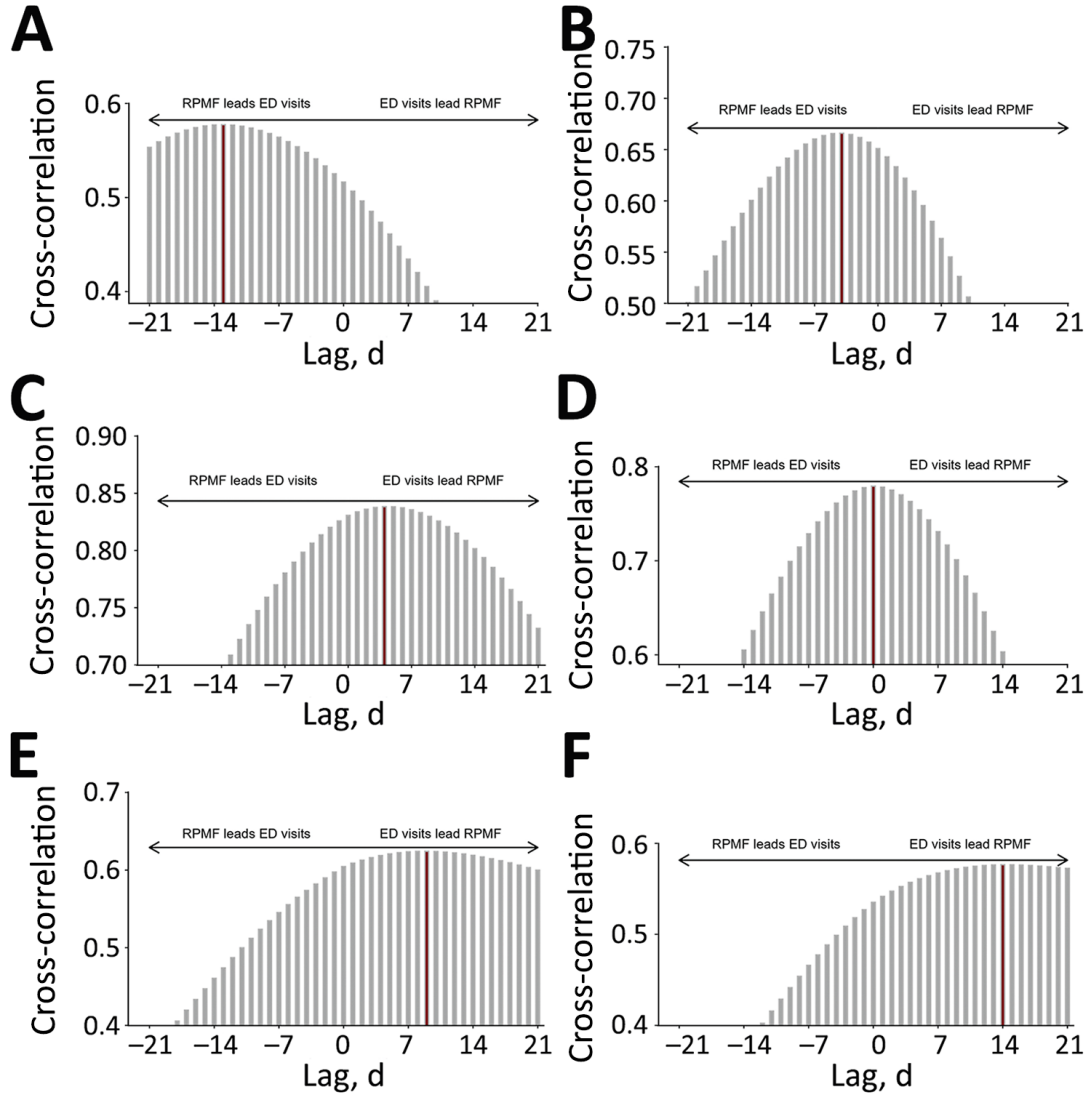
Cross-correlation between daily wastewater signals (RPMF) and National Syndromic Surveillance Program ED visit rates for SARS-CoV-2, influenza, and respiratory syncytial virus (RSV) in Harris and El Paso Counties, Texas, June 2022–June 2024. A) Harris County, SARS-CoV-2. B) El Paso County, SARS-CoV-2. C) Harris County, influenza. D) El Paso County, influenza. E) Harris County, RSV. F) El Paso County, RSV. Wastewater RPMF time series were shifted from −21 to +21 days relative to National Syndromic Surveillance Program ED visits, with Pearson correlation coefficients calculated at each daily lag. Negative lags indicate wastewater signals leading clinical visits. ED, emergency department; RPMF, reads per million filtered.

**Figure 4:**
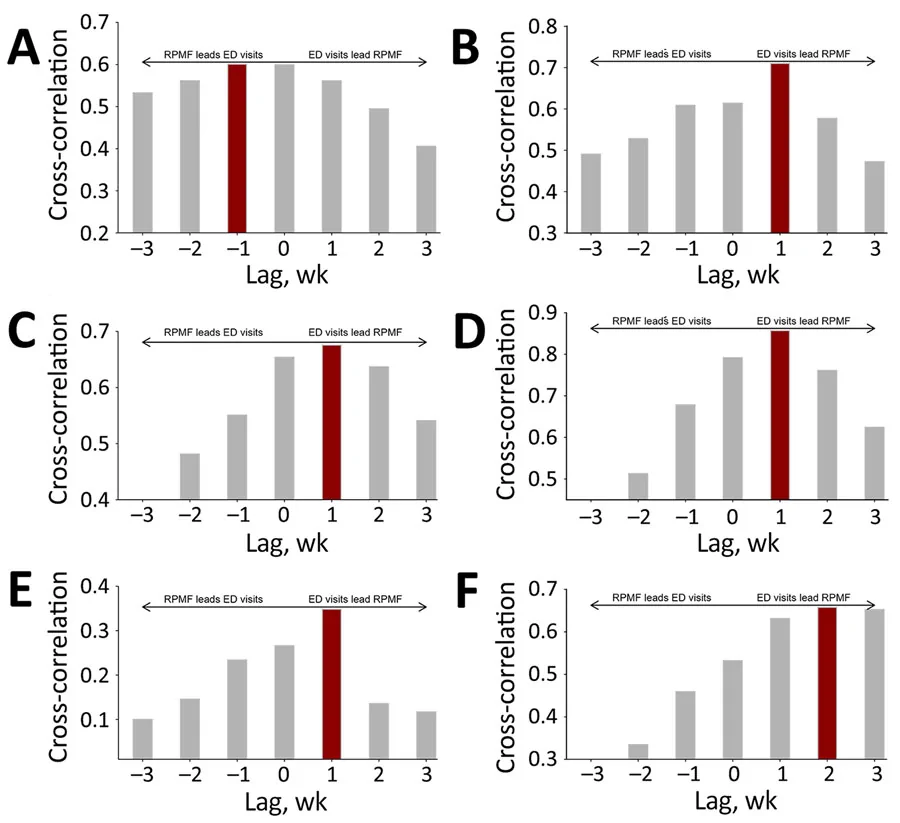
Cross-correlation between weekly wastewater signals (RPMF) and Texas All-Payer Claims Database ED claims for SARS-CoV-2, influenza, and RSV in Harris and El Paso Counties, Texas, from July 2022–June 2024. A) Harris County, SARS-CoV-2. B) El Paso County, SARS-CoV-2. C) Harris County, influenza. D) El Paso County, influenza. E) Harris County, RSV. F) El Paso County, RSV. Wastewater RPMF time series were shifted from −3 to +3 weeks relative to Texas All-Payer Claims Database claims, with Pearson correlation coefficients calculated at each weekly lag. ED, emergency department; RPMF, reads per million filtered.

We identified the lag and lead intervals by using cross-correlation analysis yielding maximum correlation coefficients (*r*_max_) for each pathogen (Figures 3, 4). SARS-CoV-2 wastewater signals generally preceded clinical metrics, with leads ranging from 3–15 days for NSSP ED visits and 1 week for TX-APCD claims. Of note, influenza patterns were more synchronous, typically showing *r*_max_ at 0 or short lags (0–4 days) across counties. In contrast, RSV demonstrated the most variable lead-lag patterns between wastewater and clinical indicators; in most counties the wastewater signal lagged NSSP ED visits, trailing them by up to 2 weeks (Tables 3, 4), and aligned inconsistently with TX-APCD claims.

**Table 3:** Pearson correlation coefficients between RPMF and 2 ED visits data sources for respiratory syncytial virus during the 2022–23 season across 4 counties, daily NSSP ED visit rates and weekly TX-APCD ED claims rates, Texas, USA*

County	RPMF versus NSSP ED visit				RPMF versus TX-APCD ED claims		
	Original *r*	Optimal lag, d	*r*_max_		Original *r*	Optimal lag, wk	*r*_max_
El Paso	0.56	15	0.61		0.34	2	0.51
Fort Bend	0.61	10	0.64		0.22	3	0.41
Harris	0.57	15	0.75		0.31	6	0.43
Lubbock	0.22	−7	0.24		0.31	6	0.49

*Optimal lag is reported relative to the clinical indicator. A negative value indicates that the wastewater signal leads the clinical indicator; a positive value indicates that it lags. The table shows the correlation at 0 lag (original), the optimal shift required to maximize the association (lag), and the resulting *r*_max_. ED, emergency department; NSSP, National Syndromic Surveillance Program; *r*_max_, maximum correlation; RPMF, reads per million filtered; TX-APCD, Texas All-Payer Claims Database.

**Table 4:** Pearson correlation coefficients between RPMF and 2 ED visits data sources for respiratory syncytial virus during the 2023–24 season across 10 counties, daily NSSP ED visit rates and weekly TX-APCD ED claims rates, Texas, USA*

County	RPMF versus NSSP ED visit				RPMF versus TX-APCD ED claims		
	Original *r*	Optimal lag, d	*r*_max_		Original *r*	Optimal lag, wk	*r*_max_
Cameron	0.56	15	0.83		−0.14	−2	0.44
Chambers	0.64	0	0.64		0.33	4	0.47
El Paso	0.82	6	0.84		0.58	1	0.69
Fort Bend	0.49	15	0.7		0.37	4	0.62
Harris	0.77	13	0.81		0.38	2	0.4
Hays	0.77	12	0.89		0.62	0	0.62
Lubbock	0.95	2	0.96		0.83	0	0.83
Travis	0.72	15	0.87		0.74	1	0.78
Wichita	0.85	3	0.87		0.17	−1	0.49
Williamson	0.65	15	0.81		0.49	4	0.78

*Optimal lag is reported relative to the clinical indicator. A negative value indicates that the wastewater signal leads the clinical indicator; a positive value indicates that it lags. The table shows the correlation at 0 (original), the optimal shift required to maximize the association (lag), and the resulting *r*_max_. ED, emergency department; NSSP, National Syndromic Surveillance Program; *r*_max_, maximum correlation; RPMF, reads per million filtered; TX-APCD, Texas All-Payer Claims Database.

Across the 10 study counties (Tables 3, 4, 5, 6, 7, 8; Appendix Figure), influenza consistently demonstrated the strongest correlations with clinical data; influenza demonstrated 0-day optimal lags relative to NSSP visits in counties such as El Paso and Fort Bend (Tables 7, 8). In contrast, SARS-CoV-2 and RSV displayed greater heterogeneity; SARS-CoV-2 typically led NSSP visits by 3–15 days; *r*_max_ values reached 0.87 in Cameron County and 0.85 in Harris County during the EG.5, HV.1, and JN.1 SARS-CoV-2 variants period and remaining more moderate in Harris County (*r*_max_ = 0.58) during the XBB period. RSV demonstrated the longest and most variable lead-lag patterns between wastewater and clinical indicators; in most counties, the wastewater signal lagged NSSP visits, with optimal lags of <15 days (Tables 3, 4), and the alignment between wastewater signals and TX-APCD claims was weak and inconsistent. For example, the contemporaneous (0-lag) correlation between wastewater and RSV claims ranged from weakly negative in Cameron County (*r* = −0.14) to only modestly positive in Wichita County (*r* = 0.17) and improved only slightly after temporal shifting.

**Table 5:** Pearson correlation coefficients between RPMF and 2 ED visit data sources for SARS-CoV-2 during the XBB variant period across 4 counties, daily NSSP ED visit rates and weekly TX-APCD ED claims rates, Texas, USA, 2024*

County	RPMF versus NSSP daily ED visit				RPMF versus TX-APCD ED claims		
	Original *r*	Optimal lag, d	*r*_max_		Original *r*	Optimal lag, wk	*r*_max_
El Paso	0.72	−5	0.74		0.62	1	0.73
Fort Bend	0.55	−3	0.56		0.7	0	0.7
Harris	0.57	−5	0.58		0.65	0	0.65
Lubbock	0.79	4	0.8		0.61	1	0.66

*Optimal lag is reported relative to the clinical indicator. A negative value indicates that the wastewater signal leads the clinical indicator; a positive value indicates that it lags. The table shows the correlation at 0 (original), the optimal shift required to maximize the association (lag), and the resulting *r*_max_. ED, emergency department; NSSP, National Syndromic Surveillance Program; *r*_max_, maximum correlation; RPMF, reads per million filtered; TX-APCD, Texas All-Payer Claims Database.

**Table 6:** Pearson correlation coefficients between RPMF and 2 ED visit data sources for SARS-CoV-2 during the combined EG.5, HV.1, and JN.1 SARS-CoV-2 variant period across 10 counties, daily NSSP ED visit rates and weekly TX-APCD ED claims rates, Texas, USA, 2024

County	RPMF versus NSSP ED visit				RPMF versus TX-APCD ED claims		
	Original *r*	Optimal lag, d	*r*_max_		Original *r*	Optimal lag, wk	*r*_max_
Cameron	0.86	−4	0.87		0.5	0	0.5
Chambers	0.43	−14	0.56		0.22	2	0.35
El Paso	0.67	0	0.67		0.36	1	0.5
Fort Bend	0.75	−3	0.76		0.61	1	0.62
Harris	0.65	−15	0.85		0.49	−2	0.51
Hays	0.71	−11	0.8		0.62	0	0.62
Lubbock	0.72	−8	0.74		0.35	0	0.35
Travis	0.75	−9	0.81		0.63	0	0.63
Wichita	0.26	7	0.27		0.32	1	0.41
Williamson	0.77	−11	0.84		0.54	−1	0.57

*Optimal lag is reported relative to the clinical indicator. A negative value indicates that the wastewater signal leads the clinical indicator; a positive value indicates that it lags. The table shows the correlation at 0 (original), the optimal shift required to maximize the association (lag), and the resulting *r*_max_. ED, emergency department; NSSP, National Syndromic Surveillance Program; *r*_max_, maximum correlation; RPMF, reads per million filtered; TX-APCD, Texas All-Payer Claims Database.

**Table 7:** Pearson correlation coefficients between RPMF and 2 ED visits data sources for influenza during the 2022–23 season across 4 counties, daily NSSP ED visit rates and weekly TX-APCD ED claims rates, Texas, USA*

County	RPMF versus NSSP ED visit				RPMF versus TX-APCD ED claims		
	Original *r*	Optimal lag, d	*r*_max_		Original *r*	Optimal lag, wk	*r*_max_
El Paso	0.98	0	0.98		0.79	1	0.87
Fort Bend	0.89	0	0.89		0.75	1	0.76
Harris	0.91	0	0.91		0.74	1	0.77
Lubbock	0.68	−13	0.95		0.52	−1	0.68

*Optimal lag is reported relative to the clinical indicator. A negative value indicates that the wastewater signal leads the clinical indicator; a positive value indicates that it lags. The table shows the correlation at 0 (original), the optimal shift required to maximize the association (lag), and the resulting *r*_max_. ED, emergency department; NSSP, National Syndromic Surveillance Program; *r*_max_, maximum correlation; RPMF, reads per million filtered; TX-APCD, Texas All-Payer Claims Database.

**Table 8:** Pearson correlation coefficients between RPMF and 2 ED visits data sources for influenza during the 2023–24 season across 10 counties, daily NSSP ED visit rates and weekly TX-APCD ED claims rates, Texas, USA*

County	RPMF versus NSSP ED visit				RPMF versus TX-APCD ED claims		
	Original *r*	Optimal lag, d	*r*_max_		Original *r*	Optimal lag, wk	*r*_max_
Cameron	0.86	0	0.86		0.25	2	0.46
Chambers	0.84	0	0.84		0.56	1	0.61
El Paso	0.9	0	0.9		0.88	0	0.88
Fort Bend	0.87	0	0.87		0.29	0	0.29
Harris	0.79	10	0.83		0.63	2	0.71
Hays	0.57	−15	0.66		0.45	−3	0.65
Lubbock	0.81	0	0.81		0.4	−2	0.47
Travis	0.53	−15	0.61		0.5	−3	0.54
Wichita	0.68	−14	0.9		0.76	0	0.76
Williamson	0.54	−15	0.63		0.4	−3	0.67

*Optimal lag is reported relative to the clinical indicator. A negative value indicates that the wastewater signal leads the clinical indicator; a positive value indicates that it lags. The table shows the correlation at 0 (original), the optimal shift required to maximize the association (lag), and the resulting *r*_max_. ED, emergency department; NSSP, National Syndromic Surveillance Program; *r*_max_, maximum correlation; RPMF, reads per million filtered; TX-APCD, Texas All-Payer Claims Database.

## Discussion

WBE can offer a population-level view of respiratory virus circulation that complements comprehensive clinical reporting in a changing surveillance landscape. Although WBE lacks a formal biological denominator to provide absolute prevalence, the use of normalized metrics such as RPMF enables a semiquantitative assessment of community viral load. Our study indicates that metagenomic WBE remains a highly informative tool, although we observed major heterogeneity in both correlation strength and lead-lag timing across diverse pathogens and geographies. Because wastewater data are most actionable for public health when they lead clinical indicators, we emphasize the direction of the wastewater–clinical relationship, and not only its strength.

Influenza exhibited the most consistent contemporaneous (0-lag) alignment with both NSSP ED visits and TX-APCD claims, demonstrating the highest correlation coefficients across the study region. Of note, influenza was often near-synchronous with clinical trends, showing 0-day lags in 5 of 10 counties, which likely reflects its robust seasonal dynamics. Furthermore, the generally shorter wastewater-to-ED lags for influenza compared with SARS-CoV-2 might be attributed to its shorter incubation period (*25*). In contrast, wastewater signals for RSV demonstrated the longest and most variable lag times, generally trailing NSSP ED visits by up to 2 weeks. That extended lag, coupled with inconsistencies between clinical data sources, such as RSV signals lagging NSSP visits while aligning inconsistently with TX-APCD claims, might stem from age-specific infection patterns and differences in healthcare-seeking behavior (*26*), particularly among pediatric populations who might contribute less to the wastewater aggregate (*27*); because young children shed comparatively little virus into the wastewater aggregate, such lower shedding might also dampen the wastewater signal and reduce any potential RSV lead time.

Discrepancies between NSSP ED visits and TX-APCD claims in their correlation with wastewater signals highlight the unique limitations of each clinical proxy. Although the NSSP captures encounters regardless of payer status, the TX-APCD is limited to insured populations (*6*,*19*). The lower correlations observed in Cameron County, which has the highest uninsured rate among the studied counties, suggest that socioeconomic factors and insurance coverage majorly influence clinical surveillance sensitivity. Furthermore, whereas the TX-APCD relies on confirmed ICD-10 diagnosis codes, NSSP data represents a hybrid of syndromes and diagnoses, which might introduce nonspecificity.

Our lead–lag findings are broadly consistent with the wider WBE literature (*29**,**30*). For SARS-CoV-2, a systematic review of COVID-19 wastewater studies reported lead times relative to clinical cases that are short and highly variable across settings, from roughly 0 to about 2 weeks, broadly consistent with the 3–15-day NSSP leads we observed (*28*). Direct comparisons are rarer for influenza and RSV, but the available studies likewise report short and inconsistent lead times. Surveillance in Wisconsin found that wastewater influenza A and RSV signals tracked closely with, and did not consistently precede, emergency department visits (*29*), and municipal influenza A wastewater signals have been reported to align with clinical cases only after substantial time-shifting (*30*). Our finding that influenza was near-synchronous and that RSV wastewater signals tended to lag clinical indicators is therefore consistent with that body of work and reinforces the view that metagenomic WBE is best positioned as a complementary, situational-awareness tool rather than a consistently leading early-warning system for these pathogens.

The first limitation of this study is the spatial misalignment between wastewater and clinical catchment areas. Sewershed boundaries often do not follow county lines, meaning the contributing population to a wastewater sample might not perfectly overlap with the persons captured in county-level clinical datasets. That geographic mismatch, combined with variations in sampling frequency and the lack of human-versus-animal source differentiation, might explain some of the observed variability in lead times. Second, the potential for nonhost genetic material to fluctuate on the basis of environmental factors remains an inherent limitation of metagenomic normalization. Finally, we computed all correlations we report on smoothed signals, wastewater RPMF interpolated to daily resolution with natural cubic splines that draw on both past and future observations, and clinical counts smoothed with a 7-day moving average, rather than on raw data streams. Such smoothing tends to inflate apparent correlation strength relative to unsmoothed data; for SARS-CoV-2, correlations computed on raw wastewater data are typically lower (r ≈ 0.35). Because the spline interpolation incorporates future and past observations, the lead and lag estimates reported here should be interpreted as descriptive temporal associations rather than strictly real-time predictive leads.

Future systems should prioritize the harmonization of clinical data streams and the refinement of spatial matching at a subcounty level to better account for system-level variability. However, we emphasize that WBE should be used to complement, not replace, traditional case reporting. Our results align with previous city-level studies showing that WBE can be a reliable and timely indicator of respiratory trends (*31*,*32*). 

Appendix Additional information about temporal alignment of wastewater signals with clinical indicators of respiratory illness postpandemic, Texas, USA.
