# Supplementary material for: Temporal Alignment of Wastewater Signals with Clinical Indicators of Respiratory Illness Postpandemic, Texas, USA
Source: Emerg Infect Dis. 2026 Aug;32(Suppl 1):S43–52. doi: 10.3201/eid3213.260141 (PMC13585683; doi:10.3201/eid3213.260141)
Supplement: Supplementary file 1 [file 26-0141-Techapp-s1.pdf]

Article DOI: https://doi.org/10.3201/eid3213.260141

EID cannot ensure accessibility for supplementary materials supplied by authors. Readers who have difficulty accessing supplementary content should contact the authors for assistance.

Temporal Alignment of Wastewater Signals with Clinical Indicators of Respiratory Illness Postpandemic, Texas, USA

Appendix

Wastewater sample processing

Raw wastewater samples (100–500 mL) were collected by the TexWeb in pre-labeled, leak-proof bottles from participating wastewater treatment plants in Texas. Samples were surface-decontaminated with 10% bleach and shipped overnight on ice to the Alkek Center for Metagenomics and Microbiome Research. Upon receipt, samples were barcoded, stored at 4°C, and a 50 mL aliquot was centrifuged at 3,374 × g for 10 min to separate solids from supernatant. The clarified supernatant was vacuum-filtered through ion-based cellulose membranes, and the virus-laden filter was bead-beaten in lysis buffer (1 min at 5 m/s, 1 min rest, 1 min at 5 m/s). The resulting lysate was centrifuged at 14–17k RPM for 2 min, and total nucleic acids were extracted using the Qiagen QIAamp Viral RNA Mini Kit and converted to double-stranded cDNA/DNA. Sequencing libraries were prepared from 25 ng of input using the Twist Library Preparation EF 2.0 kit, pooled at ≤16 samples (1.5 µg total), and enriched for viral targets by 16 h hybrid capture at 70°C with the Twist Comprehensive Viral Research Panel. Post-capture libraries underwent 12 PCR amplification cycles and were sequenced on an Illumina NovaSeq 6000 platform (2 × 150 bp). Raw BCL files were demultiplexed to FASTQ format using Illumina bcl2fastq, and reads were quality-filtered to remove adapters, low-quality bases, and host sequences. Viral reads were normalized to sequencing depth and expressed as Reads Per Million Filtered (RPMF) (*1*).

Wastewater data processing

RPMF values for respiratory pathogens were interpolated to daily resolution using generalized additive models (GAMs) implemented in mgcv v4.9–0 in R v4.4.056. For each county–virus time series, we evaluated three alternative spline bases for calendar time *t* (days since study start): cubic regression splines (piecewise cubic polynomials), penalized B-splines (P-splines), and thin-plate regression splines. Within each spline family, the smoothing parameter (λ) and basis dimension (*k*) were selected by minimizing the generalized cross-validation (GCV) score under the GCV.Cp criterion. After identifying the optimal λ and *k*, each model was refitted with those settings. The three tuned models were then compared using Akaike's Information Criterion (AIC), and the specification with the lowest AIC was selected for that county–virus series (*2*). The selected GAM was used to generate daily predictions of log-RPMF across the study period, which were subsequently back-transformed to the original scale with associated 95% confidence intervals.

References

1. Tisza M, Javornik Cregeen S, Avadhanula V, Zhang P, Ayvaz T, Feliz K, et al. Wastewater sequencing reveals community and variant dynamics of the collective human virome. Nat Commun. 2023;14:6878. PubMed https://doi.org/10.1038/s41467-023-42064-1
2. The Texas Epidemic Public Health Institute. SeqBoard [cited 2026 Jan 9]. https://dashboard.tephi.texas.gov/public-dashboard

Appendix Table 1. Wastewater sampling sites, associated catchment populations, and sampling frequencies.

Sampling Site	City	County	Population	Start date of reporting
Almeda Sims	Houston	Harris	104986	05/2022
Homestead	Houston	Harris	12393	06/2022
Metro Central	Houston	Harris	20890	06/2022
Park Ten	Houston	Harris	8544	06/2022
Turkey Creek	Houston	Harris	66430	06/2022
Humble West	Humble	Harris	Undocumented	05/2022
Fred Hurvey	El Paso	El Paso	78686	06/2022
Haskell	El Paso	EL Paso	87549	06/2022
John Hickerson	El Paso	El Paso	101327	06/2022
Roberto Bustamante	El Paso	El Paso	277401	06/2022
South Austin	Austin	Hays	Undocumented	07/2022
South Austin	Austin	Travis	Undocumented	07/2022
Walnut Creek	Austin	Williamson	Undocumented	07/2022
Baytown Central	Baytown	Harris	Undocumented	05/2023
Baytown West	Baytown	Chambers	Undocumented	05/2023
BRSV_TX1	Brownsville	Cameron	Undocumented	03/2023
LB_TX1, LB_TX2	Lubbock	Lubbock	Undocumented	11/2022
WICH_TX1	Wichita Fall	Wichita	Undocumented	02/2023
West Fondren Rd	Missouri City	Fort Bend	Undocumented	05/2023

Appendix Table 2. COVID-19-related NDC codes

NDC code	Drug Name
00069–0345	Paxlovid (Nirmatrelvir/Ritonavir)
00069–1085	Paxlovid (Nirmatrelvir/Ritonavir)
00069–1101	Paxlovid (Nirmatrelvir/Ritonavir)
00069–1245	Pepcid (famotidine)
00069–0175	Doxorubicin Hydrochloride
00069–2085	Morphine Sulfate Extended-Release Tablets
00069–5001	Paxlovid (Nirmatrelvir/Ritonavir)
00069–5017	Retacrit (Epoetin alfa-epbx)
00069–3045	Phendimetrazine
00069–5317	Paxlovid (Nirmatrelvir/Ritonavir)
00069–5321	Paxlovid (Nirmatrelvir/Ritonavir)

Appendix Table 3. Influenza-related NDC codes

NDC code	Drug Name
173068101	FLULAVAL
173068122	FLULAVAL
50242058301	INFLUENZA A VIRUS A/H1N1
50242086001	Influenza A Virus A/H1N1
50242086086	Influenza A Virus A/H1N1
50242087701	Influenza A Virus A/H1N1
50242087786	Influenza A Virus A/H1N1
4082205	AFLURIA
50090066200	FLUVIRIN
67296013101	FLUVIRIN
72769018103	INFLUENZA VIRUS VACCINE
42385098501	FLUARIX
42385098511	FLUARIX
42385098601	FLUARIX
42385098611	FLUARIX
42385098701	FLUARIX
42385098711	FLUARIX
93818064	Influenza Virus Vaccine
527459113	Influenza Virus Vaccine
527459213	Influenza Virus Vaccine
527459313	Influenza Virus Vaccine
527513762	Influenza Virus Vaccine
14335038001	FLUVIRIN
27241013909	INFLUENZA VIRUS VACCINE
31722010860	FLUVIRIN
33332011010	FLUCELVAX

NDC code	Drug Name
49281031710	FLULAVAL
49281031810	FLULAVAL
49281041910	FLULAVAL
49281042010	FLULAVAL
49281052110	FLULAVAL
49281052210	FLULAVAL
49281072910	FLULAVAL
49281073010	FLULAVAL
49281081110	FLULAVAL
49281081210	FLULAVAL
49281091310	FLULAVAL
49281091410	FLULAVAL
53002638701	FLUVIRIN
59651059760	INFLUENZA VIRUS VACCINE
64380079701	INFLUENZA VIRUS VACCINE
64380079801	INFLUENZA VIRUS VACCINE
64380079901	INFLUENZA VIRUS VACCINE
64380079906	INFLUENZA VIRUS VACCINE
64380079907	INFLUENZA VIRUS VACCINE
64380087975	INFLUENZA VIRUS VACCINE
67296151401	FLUVIRIN
67296164701	FLUVIRIN
67296172501	FLUVIRIN
67296206401	FLUVIRIN
67296207801	FLUVIRIN
68071256501	FLUVIRIN
68071261201	FLUVIRIN
68071275401	FLUVIRIN
68094005061	Influenza Virus Vaccine
68180067511	FLUVIRIN
68180067611	FLUVIRIN
68180067711	FLUVIRIN
68180067801	FLUVIRIN
68788760006	INFLUENZA VIRUS VACCINE
68788812201	INFLUENZA VIRUS VACCINE
68788812306	INFLUENZA VIRUS VACCINE
68788830901	INFLUENZA VIRUS VACCINE
68788832001	INFLUENZA VIRUS VACCINE

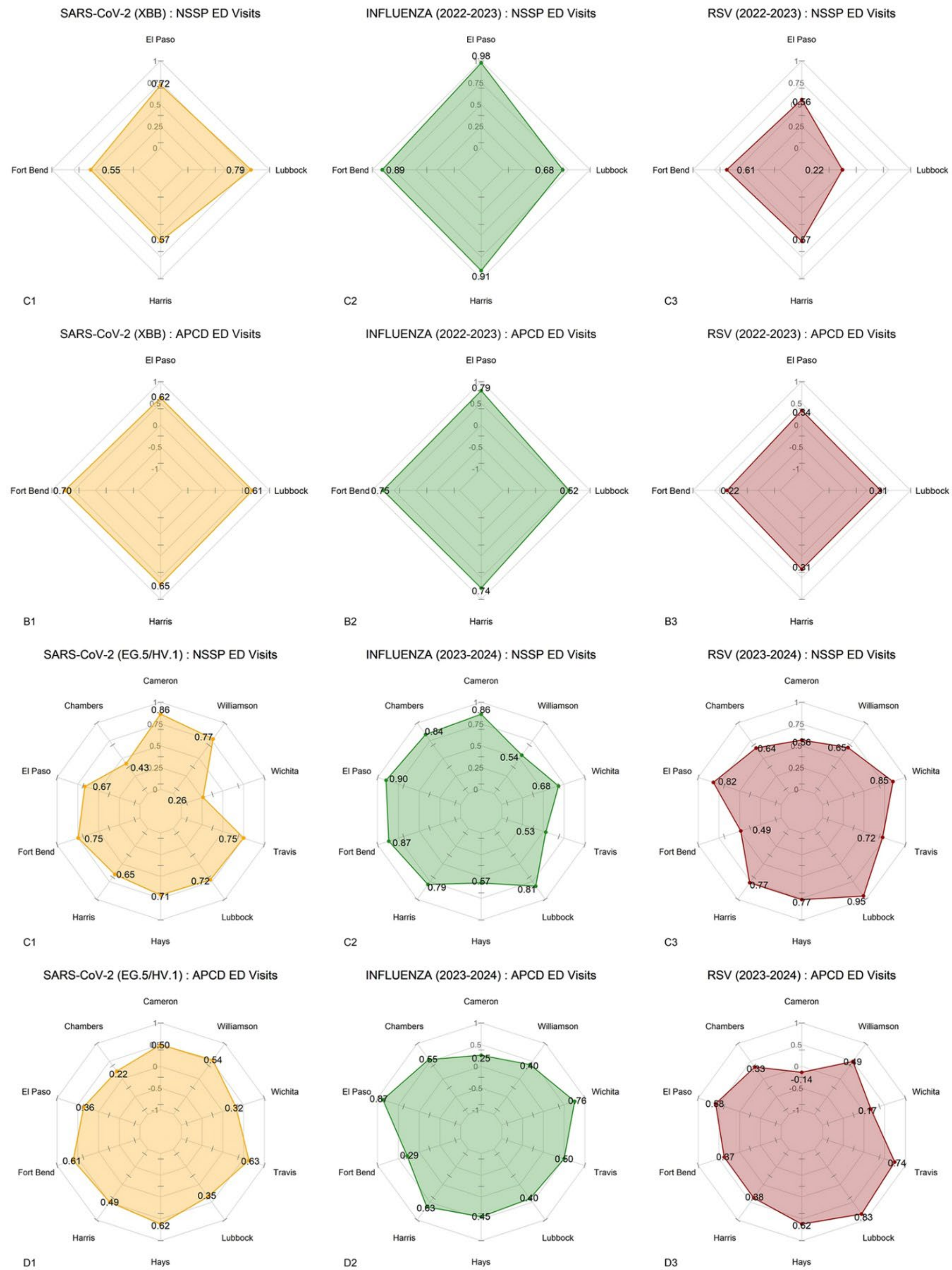

Appendix Figure. Radar plots illustrating correlation strength between wastewater sequencing (RPMF) and clinical indicators across Texas counties. Panels show Pearson correlation coefficients (*r*) for daily

wastewater RPMF versus NSSP ED visits (Rows A and C) and weekly wastewater RPMF versus TX-APCD ED claims (Rows B and D). Columns represent SARS-CoV-2 (yellow), influenza (green), and RSV (red). SARS-CoV-2 results are presented for the XBB period (A1, B1) and the EG.5/HV.1 period (C1, D1); influenza results are presented for the 2022–2023 season (A2, B2) and the 2023–2024 season(C2, D2); and RSV results are presented for the 2022–2023 season (A3, B3) and the 2023–2024 season(C3, D3). The number of counties displayed varies by panel based on data availability, with blue values indicating the correlation coefficient for each respective jurisdiction, including Cameron, Chambers, El Paso, Fort Bend, Harris, Hays, Lubbock, Travis, Wichita, and Williamson.
